# Simultaneous Disruption of Phosphate and Carbon Signaling Regulators Enables Adaptive Gene Expression Through Non-Cognate Phosphorylation of PhoP

**DOI:** 10.3390/biology15141138

**Published:** 2026-07-13

**Authors:** Jae-Yong Park, Wael Abdel-Fattah, F. Marion Hulett

**Affiliations:** 1 Molecular Biology Research Building, Department of Biological Sciences, University of Illinois, Chicago, IL 60607, USA; jaepark@cu.ac.kr (J.-Y.P.); hulett@uic.edu (F.M.H.); 2 Department of Food Science and Nutrition, Daegu Catholic University, Gyeongsan 38438, Republic of Korea

**Keywords:** *Bacillus subtilis*, carbon catabolite regulation, CcpA, non-cognate phosphorylation, PhoPR, phosphate-starvation, YycFG

## Abstract

The PhoP–PhoR two-component system (TCS) controls transcription of the Pho regulon in response to inorganic phosphate limitation in *Bacillus subtilis*. In addition to its role in phosphate homeostasis, increasing evidence suggests that central metabolic pathways influence Pho regulon activity. Here, we investigated the mechanism underlying phosphate-independent activation of the Pho regulon in a *ccpA* mutant lacking the global regulator of CCR. Gene expression analyses demonstrated strong glucose-dependent induction of Pho regulon genes in the absence of both CcpA and the cognate histidine kinase PhoR. Using PhosTag gel electrophoresis analysis and a phoasphoablative mutation in *phoP* (D53A), we detected early PhoP phosphorylation and determined that Pho regulon activation requires a phosphorylatable form of PhoP, even when the cognate histidine kinase *phoR* gene was deleted. Because PhoR can phosphorylate non-cognate response regulator YycF, we examined whether the essential YycFG signaling system contributes to PhoP activation. Co-immunoprecipitation and in vitro phosphorylation assays confirmed a direct interaction between PhoP and YycG and demonstrated in vitro PhoP phosphorylation by YycG. Taken together, we propose a model in which CcpA limits excessive PhoP accumulation to preserve signaling specificity and prevent aberrant activation through non-cognate kinases. Furthermore, our study reveals a previously unrecognized link between carbon catabolite repression and phosphate starvation signaling and demonstrates another crosstalk between two-component signal transduction systems in the Gram-positive bacterium, *B. subtilis*.

## Simple Summary

Bacteria survive nutrient limitation and antibiotic stress by activating adaptive signaling pathways. During infection, phosphate becomes scarce in intracellular niches, such as macrophages, while carbon catabolite repression (CCR) mediated by CcpA is often diminished because preferred carbon sources are limited. We investigated how phosphate starvation (Pho) signaling is maintained under these conditions when the primary phosphate-sensing pathway is disrupted. Loss of CcpA caused strong activation of phosphate response genes even in the absence of the canonical sensor kinase PhoR. We further found that the cell wall regulator YycG/WalK kinase can directly phosphorylate the Pho response regulator PhoP, revealing regulatory crosstalk that may influence bacterial persistence and antimicrobial resistance (AMR).

## 1. Introduction

To survive phosphate starvation (Pho), bacteria activate a coordinated transcriptional program that regulates genes required for phosphate acquisition and metabolic adaptation [1]. In *B. subtilis*, this response is governed by the Pho regulon, controlled by the PhoP-PhoR TCS [2]. Under phosphate limitation, the membrane-bound histidine kinase PhoR senses environmental signals and undergoes autophosphorylation at a conserved histidine residue. The phosphoryl group is subsequently transferred to a conserved aspartate residue in the response regulator PhoP, activating transcription of the *phoPR* operon and downstream Pho regulon genes involved in phosphate scavenging and stress adaptation [1]. Pho regulon genes include more than 20 genes or operons, such as *phoA* [3] and *phoB* [4], encoding alkaline phosphatases, and *phoD* [5], encoding phosphodiesterases that enable utilization of alternative phosphate sources.

Regulation of the *phoPR* operon is highly complex and integrates multiple environmental and physiological inputs. At least six promoters have been identified upstream of *phoP*, each differentially regulated according to growth phase and environmental conditions [6,7]. These promoters are recognized by distinct RNA polymerase holoenzymes, including the σ^A^-dependent promoters (P_A3_, P_A4_, P_A6_), the σ^B^-dependent promoter (P_B1_), and the σ^E^-dependent promoter (P_E2_) [6,7,8]. Promoter P_5_ is active only in a *sigB* mutant background, although its cognate sigma factor remains unknown [6]. Notably, P_A6_ is induced following inactivation of the global regulator of carbon catabolite repression (CcpA) or mutation of the *cre* (catabolite-responsive element) box within the phoP promoter region, providing direct evidence for functional coupling between CCR and Pho-responses [7]. Furthermore, several promoters (P_B1_, P_E2_, P_A4_, and P_A6_) are repressed by the global transcriptional regulator ScoC, linking phosphate sensing to developmental processes, such as sporulation [8]. In addition to its complex transcriptional regulation, the PhoP-PhoR TCS is part of a signal transduction network that consists of at least three TCSs (PhoP-PhoR, ResD-ResE, and Spo0A∼P) and the transition state regulator, AbrB [9]. The Spo0A phosphorelay governs the transition into stationary phase and sporulation, whereas the ResD-ResE TCS coordinates aerobic and anaerobic respiratory metabolism [10,11,12,13]. Together, these multilayered regulatory mechanisms demonstrate that the PhoP-PhoR TCS is embedded within broader metabolic, stress-response, and developmental networks that enable bacterial adaptation to fluctuating environmental conditions.

Under specific stress conditions, controlled cross-regulation between non-cognate TCSs may provide adaptive advantages by integrating multiple signals and enabling survival in fluctuating environments [14]. Previous in vitro studies frequently detect non-cognate phosphorylation in TCSs; such interactions are generally suppressed in vivo by sensory kinase phosphatase activity and kinetic insulation [15]. However, because histidine kinases and response regulators are structurally conserved across TCSs, signaling crosstalk between pathways is mechanistically possible [16]. In *B. subtilis*, reciprocal interactions between the PhoP–PhoR and the essential YycF–YycG (WalR-WalK) TCSs have been reported [17]. First, *yocH*, encoding a putative autolysin, is induced during phosphate starvation in a YycF-dependent but PhoP-independent manner. Moreover, *yocH* transcription is abolished in *phoR* mutants, and in vitro studies have shown that PhoR directly phosphorylates YycF, suggesting functional crosstalk between phosphate sensing and cell wall regulatory networks.

In this study, we identified physiological conditions under which PhoP undergoes phosphorylation in vivo independently of its cognate kinase PhoR and ambient phosphate availability and demonstrated in vitro that YycG directly phosphorylates PhoP through a specific PhoP–YycG interaction. These findings uncover an alternative mechanism of PhoP activation that may enhance bacterial adaptation within host infection niches, with important implications for the regulation of antimicrobial resistance, virulence, and persistence when canonical PhoR-dependent signaling is compromised.

## 2. Materials and Methods

### 2.1. Strains and Plasmids

Bacterial strains, plasmids, and primers used in this study are listed in Table 1. Stocks stored at −80 °C were streaked onto Luria–Bertani (LB) agar (Thermo Scientific™ Invitrogen™, Hanover Park, IL, USA) for Escherichia coli or tryptose blood agar base (Thermo Scientific™ Oxoid™, Hanover Park, IL, USA) supplemented with 0.5% glucose (Merck, Westchester, IL, USA) for *B. subtilis*. Selective antibiotics (Merck, Westchester, IL, USA) were used at the following final concentrations: chloramphenicol (Cm, 1 mg/L), tetracycline (Tet, 10 mg/L), kanamycin (Kan, 10 mg/mL), spectinomycin (Spc, 100 mg/L), erythromycin (Em, 5 mg/L), and ampicillin (Amp, 200 mg/L).

Isogenic mutant strains carrying promoter–*lacZ* fusions (*phoP40-lacZ*, *phoB*-P_V_-*lacZ*, *phoA-lacZ*, and *phoD-lacZ*) in *phoR, ccpA*, or double mutant backgrounds were constructed by natural transformation using chromosomal DNA from previously described strains [18,19,20,21]. Transformants were selected on appropriate antibiotics (Tet or Spc). Double mutants were generated by sequential transformation and selection as described in the strain construction scheme.

Site-directed mutagenesis was used to generate non-phosphorylatable PhoP variants (*phoP* D53A) using the QuickChange II system (Agilent, Arlington Heights, IL, USA). Mutant constructs were verified by sequencing. The *phoR* deletion allele was generated by insertion of a kanamycin resistance cassette from *Streptococcus faecalis* (*aphA3*) into a *BalI*-digested *phoR* locus [28], and confirmed by antibiotic selection and molecular screening.

### 2.2. Growth Media and Culture Conditions

All components of growth media were from Merck, Westchester, IL, USA unless otherwise specified. Low-phosphate complex medium (LPCM) was used for qualitative Pho regulon induction assays. LPCM contained ammonium acetate (3 g/L), MgSO_4_ (0.25 g/L), calcium acetate (0.02 g/L), MnCl_2_ (0.5 mM), Bacto peptone (10 g/L), amino acid mixture (0.05 mg/mL), vitamins, and 1.5% agar, buffered with 50 mM Tris (pH 6.9). Where indicated, high-phosphate medium (HPCM) was supplemented with 10 mM K_2_HPO_4_/KH_2_PO_4_ (pH 7.0). Glucose was added at 2% (w/v) in LPCMG and HPCMG variants. X-gal (Merck, Westchester, IL, USA). was used at 100 mg/L for promoter activity visualization.

For quantitative assays, modified LPDM (low-phosphate defined medium) and HPDM (high-phosphate defined medium) were used as previously described [29], with modifications including omission of CoCl_2_, adjustment of ZnCl_2_ to 0.3 mM, and replacement of fructose with 2% glucose. Where indicated, media were supplemented with glutamate (0.8%) and branched-chain amino acids (0.05 mg/mL).

### 2.3. Enzyme Activity Assays

Alkaline phosphatase (APase) activity was measured using p-nitrophenyl phosphate (Merck, Westchester, IL, USA, at 0.1 M in 1 M CHES, pH 9.5). Reactions were incubated at 37 °C, and absorbance was recorded at 420 nm. One unit of APase activity corresponds to 1 µmol p-nitrophenol released per minute and was normalized to OD_540_.

β-galactosidase activity was measured using o-nitrophenyl-β-D-galactopyranoside (ONPG, Merck, Westchester, IL, USA) according to Ferrari et al. [30]. One unit corresponds to 0.33 nmol o-nitrophenol released per minute and was normalized to total protein content. Protein concentrations were estimated as previously described [5].

### 2.4. Protein Expression and Purification

His_10_-PhoP, GST-PhoR (N-terminally truncated), and His_6_-YycG cytoplasmic domain (YycGc) were expressed in *E. coli* BL21(DE3) or BL21(DE3)/pLysS using plasmids listed in Table 1. Induction and purification were performed as previously described [22,23,24] using affinity chromatography under native conditions. Protein purity was assessed by SDS-PAGE.

### 2.5. Western Blot Analysis

All chemicals and enzymes used in cell lysis and Western blot analysis were from Merck, Westchester, IL, USA unless otherwise specified. PhoP detection was performed as described previously [31] with modified lysis buffer containing Tris-HCl (50 mM, pH 7.0), EDTA (10 mM), lysozyme (15 mg/mL), DNase I (10 µg/mL), RNase A (100 µg/mL), and protease inhibitors. Proteins were transferred to PVDF membranes (MilliporeSigma™ Immobilon™, Fischer Scientific, University of Illinois at Chicago, Chicago, IL, USA) at 100 V using a Bio-Rad wet-blot transfer system (Des Plaines, IL, USA). Anti-PhoP CTD antibodies (Biologic Resources Laboratory, University of Illinois at Chicago, Chicago, IL, USA) were used at 1:4000 dilution, and HRP-conjugated secondary antibodies (Bio-Rad, Des Plaines, IL, USA) at 1:25,000 dilution. Detection was performed using chemiluminescence (SuperSignal West Pico, Thermo Fisher, Hanover Park, IL, USA).

### 2.6. In Vitro Phosphorylation Assays

All chemicals and enzymes used in cell lysis and Western blot analysis were from Merck, Westchester, IL, USA unless otherwise specified. Autophosphorylation and phosphotransfer reactions were performed in HEPES buffer (50 mM, pH 8.0) containing KCl (50 mM) and MgCl_2_ (50 mM). HK proteins were incubated with γ-^32^P-ATP (PerkinElmer, Chicago, IL, USA) and purified using PD SpinTrap G-25 columns (Bio-Rad, Des Plaines, IL, USA) prior to phosphotransfer to PhoP. Reactions were analyzed using Phos-tag SDS-PAGE (25 µM Phos-tag acrylamide, MnCl_2_ 50 µM from FUJIFILM Wako Chemicals, Japan) followed by autoradiography and phosphorimaging.

### 2.7. In Vivo Phosphorylation Analysis

All chemicals and enzymes used in in vitro phosphorylation analysis were from Merck, Westchester, IL, USA unless otherwise specified. Cells were grown in LPDMglu and harvested at OD_540_ = 0.5. To minimize the risk of phosphate group hydrolysis during sample preparation, cell pellets were stabilized in RNAlater (Tribioscience™, Thermo Scientific™, Hanover Park, IL, USA) and lysed in EDTA-free Lysis buffer (50 mM Tris-HCl (pH 7.8), 150 mM NaCl, 1% Triton X-100, 1mg/mL lysozyme) containing freshly prepared protease inhibitor (1 mM PMSF), and phosphatase inhibitor mix (1 mM sodium orthovanadate and 20 mM sodium fluoride). The resulting lysates were analyzed by Phos-tag SDS-PAGE without prolonged sample handling. Phosphorylated and unphosphorylated PhoP bands were subsequently quantified using an image processing and analysis program (ImageJ from NIH, https://imagej.net/nih-image/index.html).

### 2.8. Co-Immunoprecipitation

All chemicals used in cross-linking and quenching were from Merck, Westchester, IL, USA. Cells of the *phoR ccpA* mutant strain were grown in HPDMglu medium to mid-exponential phase (OD_540_ = 0.5). Cultures were crosslinked with 1% formaldehyde for 15 min, and the reaction was quenched with glycine (to final concentration of 0.2 M). Cells were subsequently harvested, washed three times, and lysed by French press disruption.

Co-immunoprecipitation was performed using anti-PhoP CTD antibodies and the Pierce Co-Immunoprecipitation Kit (Thermo Fisher Scientific, Hanover Park, IL, USA) according to the manufacturer’s instructions. Immunoprecipitated protein complexes were separated by SDS-PAGE and analyzed by immunoblotting using anti-YycG antibodies (kindly provided by the laboratory of James A. Hoch). Following detection, membranes were stripped using glycine buffer (25 mM, pH 2.0), washed three times, and reprobed with anti-PhoP CTD antibodies.

### 2.9. In Vitro Phosphotransfer Between Histidine Kinases and PhoP

Phosphorylated PhoR or YycG cytoplasmic domains were incubated with PhoP (400 nM). Time-course samples were collected, quenched with SDS loading buffer, and analyzed by SDS-PAGE. Radioactive signals were detected using a phosphorimager system (Azure Biosystems, Dublin, CA, USA).

### 2.10. Statistical Analysis and Biological Replicates

**Figure 1:**
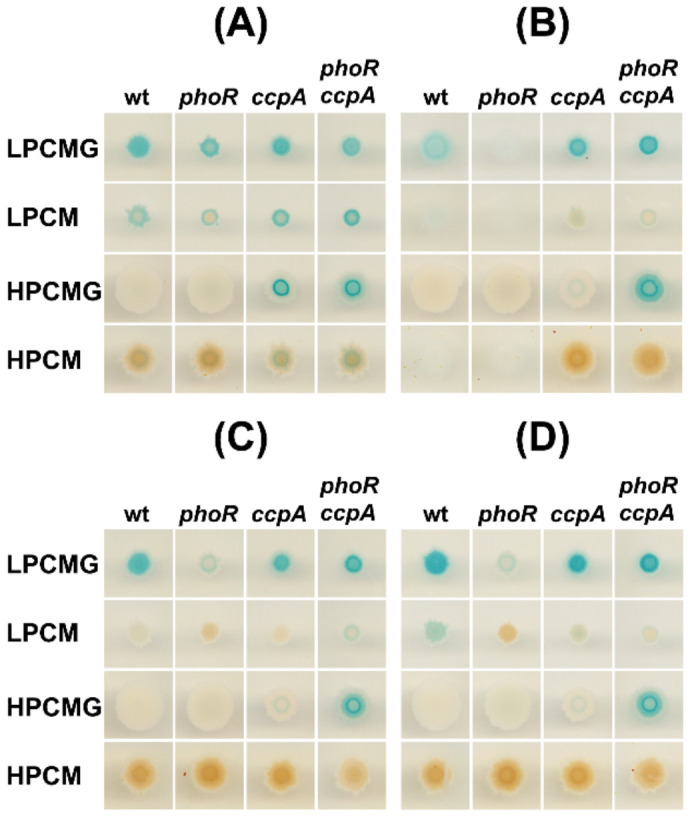
Expression of Pho regulon genes in wild-type and mutant backgrounds. (**A**) Expression of the full-length *phoP* promoter–*lacZ* fusion (*phoP40-lacZ*). (**B**) Expression of the *phoB*-P_V_-*lacZ* fusion. *phoB* (formerly *phoAIII*) encodes alkaline phosphatase B (APaseB; formerly APaseIII). P_V_ denotes the σ^A^-dependent vegetative promoter of *phoB*. (**C**) Expression of the *phoA*-*lacZ* fusion. *phoA* (formerly *phoAIV*) encodes alkaline phosphatase A (APaseA; formerly APaseIV). (**D**) Expression of the *phoD*-*lacZ* fusion. *phoD* encodes the phosphodiesterase PhoD. For plate assays, 2 μL aliquots of cell suspensions containing approximately 1 × 10^7^ cells of wild-type or *B. subtilis* mutant strains carrying Pho regulon reporter fusions (*phoB*-P_V_-*lacZ*, *phoA*-*lacZ*, or *phoD*-*lacZ*) or the autoregulated *phoP40-lacZ* fusion were spotted onto LPCMG (low-phosphate complex medium containing glucose), LPCM (low-phosphate complex medium lacking glucose), HPCMG (high-phosphate complex medium containing glucose), and HPCM (high-phosphate complex medium lacking glucose) agar plates supplemented with X-Gal. Plates were incubated at 37 °C for 48 h.

**Figure 2:**
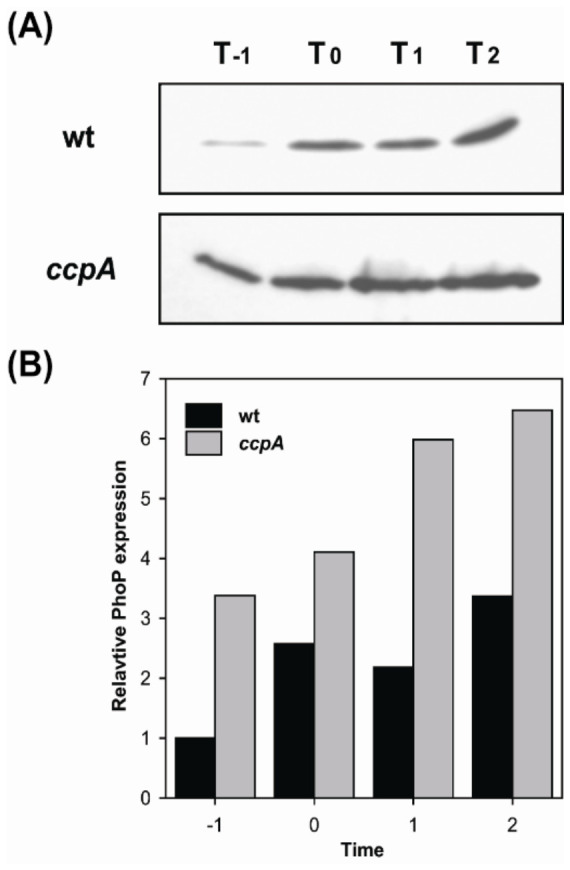
Expression of PhoP in the wild-type strain and the *ccpA* mutant during growth in LPDMglu. (**A**) Western blot analysis of PhoP levels in the wild-type strain and the *ccpA* mutant. (**B**) Relative PhoP expression levels normalized to the PhoP level of the wild-type strain at T_−1_. For detection of intracellular PhoP levels, samples were collected at the indicated time points during growth in LPDMglu medium (T_0_) denotes the transition state). Following microscopic cell counting, aliquots corresponding to 5 × 10^7^ cells were subjected to Western blot analysis as described in Materials and Methods. Two biological replicates confirmed the increased expression levels of PhoP in the *ccpA* mutant compared to wild-type in exponentially growing cells. The original Western blot images are summarized in File S1.

**Figure 3:**
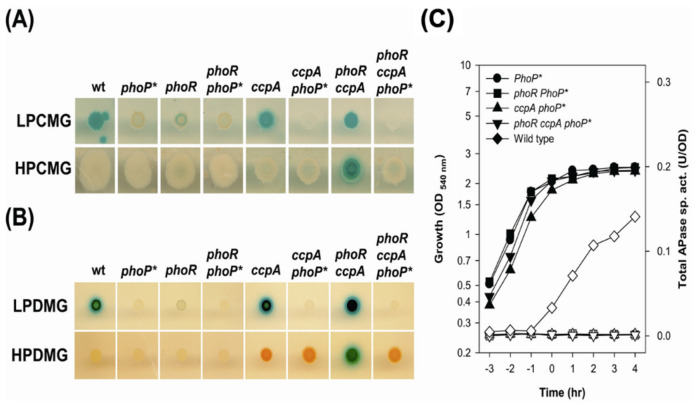
Expression of *phoB*-P_V_-*lacZ* in strains carrying the non-phosphorylatable PhoP D53A mutant protein. (**A**) Expression of *phoB*-P_V_-*lacZ* on LPCMG and HPCMG plates. (**B**) Expression of *phoB*-P_V_-*lacZ* on LPDM and HPDM plates. LPDM and HPDM are low- and high-phosphate defined media, respectively, supplemented with glutamate and branched-chain amino acids (BCAAs) as required for growth. phoP* denotes strains carrying the non-phosphorylatable PhoP D53A allele. *B. subtilis* strains were inoculated and cultured as described in Figure 1. (**C**) Growth curves (closed symbols) and total alkaline phosphatase (APase) specific activities (open symbols) of strains carrying non-phosphorylatable PhoP D53A during growth in LPDMglu. Circles, squares, triangles, and inverted triangles represent wild-type, *phoR*, *ccpA*, and *phoR ccpA* mutant strains containing PhoP D53A (PhoP*), respectively. Diamonds represent a wild-type strain carrying the native *phoP* allele and were included as a positive control.

**Figure 4:**
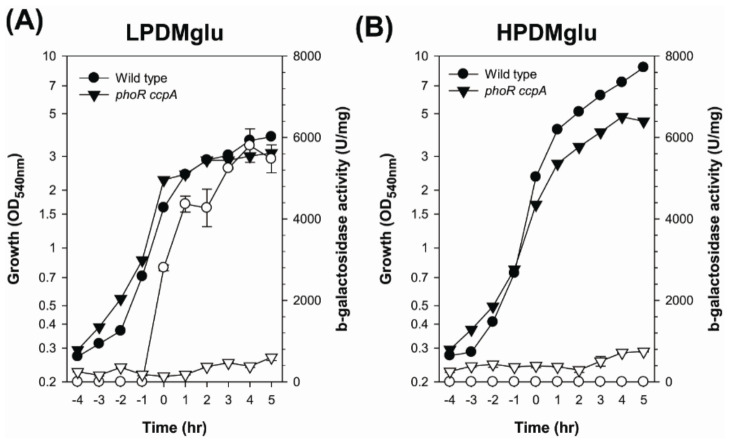
Temporal induction of *phoB*-P_V_-*lacZ* expression in wild-type and *phoR ccpA* mutant strains. (**A**) Expression during growth in LPDMglu. (**B**) Expression during growth in HPDMglu. Growth (closed symbols) and β-galactosidase activities (open symbols). Data points represent the mean values of three independent experiments, and error bars indicate the standard deviation.

**Figure 5:**
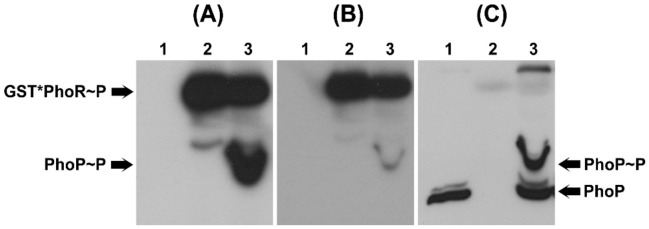
Separation of PhoP and phosphorylated PhoP (PhoP~P) using Phos-tag^TM^ acrylamide SDS–PAGE and γ−^32^P-ATP. Following phosphotransfer from autophosphorylated GST-*PhoR~P to PhoP, proteins were separated by Phos-tag^TM^ acrylamide SDS–PAGE, and the gel was exposed to X-ray film for 18 h (**A**). Proteins were subsequently transferred onto a PVDF membrane, which was exposed to X-ray film for 4 h (**B**). Western blot analysis was then performed using an antibody specific for the PhoP C-terminal domain (PhoP CTD) (**C**). Lane 1, PhoP; lane 2, autophosphorylated GST-*PhoR; lane 3, autophosphorylated GST-*PhoR incubated with PhoP. The original Western blot images are summarized in File S1.

**Figure 6:**
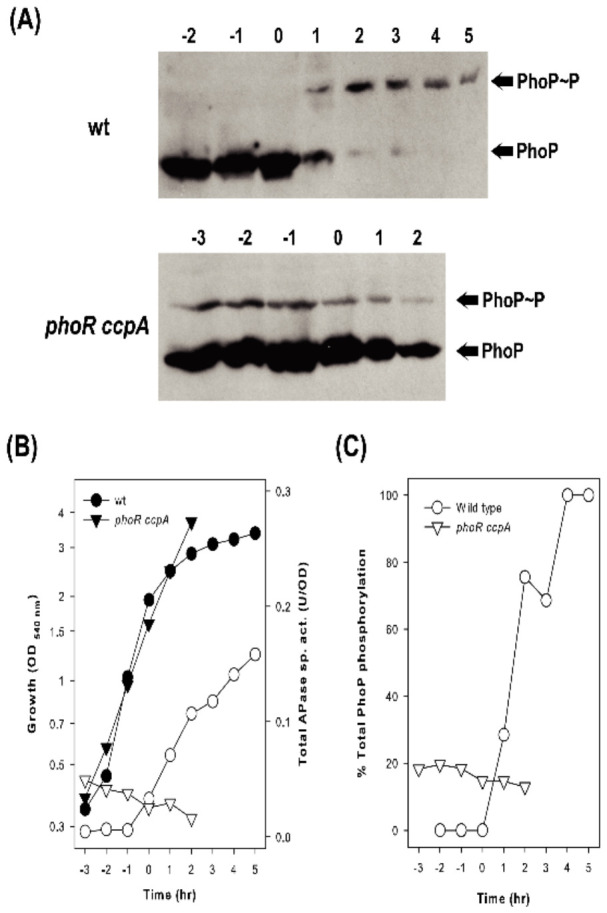
In vivo phosphorylation state of PhoP in wild-type and *phoR ccpA* strains. (**A**) Detection of in vivo phosphorylated PhoP during growth in LPDMglu using Phos-tag^TM^ acrylamide SDS–PAGE combined with Western blot analysis. (**B**) Growth curves (closed symbols) and total alkaline phosphatase (APase) specific activities (open symbols) in LPDMglu. Circles represent the wild-type strain and inverted triangles represent the *phoR ccpA* mutant. (**C**) Relative proportion of PhoP~P expressed as a percentage of total PhoP in wild-type and *phoR ccpA* strains. The original images are summarized in File S1.

**Figure 7:**
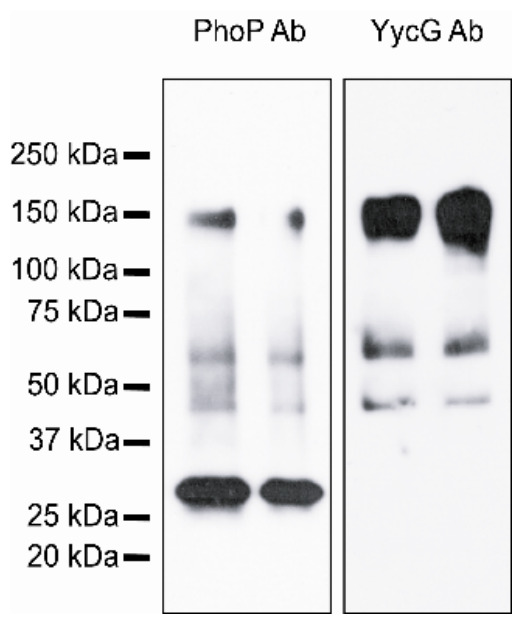
Co-immunoprecipitation of YycG with PhoP in the *phoR ccpA* mutant strain. Exponentially growing *phoR ccpA* mutant cells (OD_540_ = 0.5) were subjected to chemical crosslinking followed by immunoprecipitation of cell extracts using anti-PhoP CTD antibodies, as described in Section 2. PhoP (left panel) and YycG (right panel) were detected by Western blot analysis using anti-PhoP CTD and anti-YycG antibodies, respectively. The original images are summarized in File S1.

**Figure 8:**
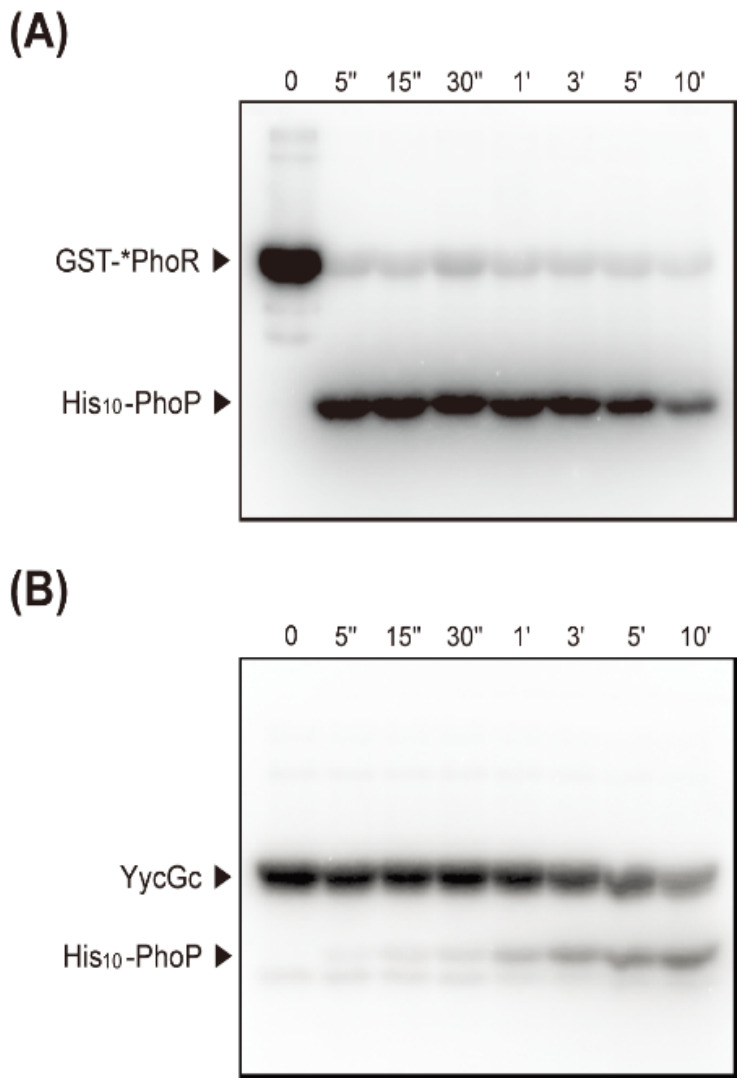
In vitro phosphotransfer between GST-PhoR or YycG_C_ and His_10_-PhoP. Samples were collected at the indicated time points from 5 s (5”) to 10 min (10’), denatured, and separated by SDS–PAGE. The 0 min samples in A and B serve as negative controls of phosphotransfer reactions and correspond to the autophosphorylated kinases without the response regulator PhoP, whereas PhoP lacks any autophosphorylation activity, as shown in lane 1 in Figure 5A–C. Gels were dried and visualized using a phosphorimager. (**A**) Phosphotransfer between GST-*PhoR (a GST-tagged N-terminal truncated soluble form of PhoR kinase) and His_10_-PhoP. (**B**) Phosphotransfer between YycG_C_ (the cytoplasmic region of YycG fused to a 6 × His tag) and His_10_-PhoP.

Drop plate assays were repeated in at least three independent experiments using different serial dilutions, and representative images are shown in Figures 1 and 3. Alkaline phosphatase (APase) and β-galactosidase activity assays (Figures 4 and 6) were performed in at least three independent biological replicates, and the data are presented as the mean values with error bars for standard deviation. Western blotting and co-immunoprecipitation experiments (Figures 2, 6 and 7) were independently repeated using biological replicates to confirm reproducibility, and representative blots are presented. In vitro phosphorylation assays (Figures 5 and 8) were independently repeated under identical reaction conditions using a fixed reaction time to confirm reproducibility. Representative results from both fixed-time for Figure 5 and time-course for Figure 8 phosphorylation assays are shown.

## 3. Results

### 3.1. PhoR-Independent Induction of the Pho Regulon in a ccpA Mutant Background

Previous studies showed that expression of the *phoB*-P_V_–*lacZ* fusion in LPCM requires the presence of a preferred carbon source, such as glucose (LPCMG). Moreover, under these conditions, *phoB*-P_V_–*lacZ* is expressed in a *phoR ccpA* double mutant in a PhoP-dependent manner, despite the absence of the cognate histidine kinase PhoR [32]. To determine whether this PhoR-independent activation is a general feature of Pho regulon genes, we analyzed additional Pho-regulated promoter–*lacZ* fusions (*phoA*, *phoB-*P_V_, and *phoD*) in wild-type, *phoR, ccpA*, and *phoR ccpA* strains grown on low- or high-phosphate complex media with or without glucose (LPCM, HPCM; LPCMG, HPCMG) (Figure 1). In LPCMG, all three fusions showed strong β-galactosidase activity in wild-type, *ccpA*, and *phoR ccpA* strains, whereas the *phoR* mutant exhibited minimal or no activity, indicating that PhoR is not required for Pho regulon induction in a *ccpA* mutant background. In contrast, under LPCM conditions, all strains displayed only basal expression levels, consistent with the requirement for a preferred carbon source for Pho regulon activation.

Notably, under HPCMG conditions, the *phoA*, *phoB*-P_V_, and *phoD* fusions were strongly induced exclusively in the *phoR ccpA* double mutant, while remaining uninduced in wild-type, *ccpA*, and *phoR* single mutants. No expression was detected under HPCM in any genetic background. These data indicate that PhoR-independent activation of Pho regulon genes in the *ccpA* background is dependent on the presence of glucose but is largely independent of extracellular phosphate availability. Consistent with these findings, the *phoP40–lacZ* fusion exhibited similar expression patterns across media and genetic backgrounds (Figure 1A). The *phoR* mutant showed consistently low activity under all conditions, whereas expression was markedly increased in *ccpA* and *phoR ccpA* strains under HPCMG. This observation aligns with previous reports demonstrating that loss of CcpA relieves repression of the PhoP promoter P_A6_ in the presence of glucose, independently of phosphate concentration [7].

### 3.2. Pho Regulon Gene Induction Depends Not on PhoP Abundance but on PhoP Phosphorylation

Because both phosphorylated and non-phosphorylated PhoP can support transcription from Pho-dependent promoters in vitro, we hypothesized that increased PhoP abundance in a *ccpA* mutant background might be sufficient to activate Pho regulon genes in the absence of phosphorylation. In vitro transcription studies using the *phoB*-P_V_ promoter showed that approximately 16-fold higher concentrations of non-phosphorylated PhoP are required to achieve ~85% of the transcriptional activity of phosphorylated PhoP [31]. Consistently, in vivo derepression of the PhoP promoter P_A6_ in a *ccpA* mutant leads to a 3-fold or greater increase in *phoP*–*lacZ* expression compared with wild-type, whereas no detectable expression is observed in the wild-type background [7].

To determine whether PhoP abundance is increased in the *ccpA* mutant, quantitative Western blot analysis was performed during growth in low-phosphate defined medium supplemented with glutamate and branched-chain amino acids (LPDMglu), conditions required to compensate for the growth defect of the *ccpA* mutant due to impaired expression of the ilv-leu operon and *gltAB* [33,34]. PhoP protein levels were significantly elevated in the *ccpA* mutant compared with wild-type, with fold differences varying across growth phases (Figure 2).

To test whether increased PhoP abundance is sufficient for Pho regulon induction, a non-phosphorylatable PhoP variant (PhoP D53A) was constructed and introduced into wild-type, *phoR*, *ccpA*, and *phoR ccpA* backgrounds. Pho regulon activity was assessed using *phoB*-P_V_–*lacZ* reporter fusions on complex and defined media plates (LPCMG, HPCMG, LPDM, HPDM) and by alkaline phosphatase assays in LPDMglu. Across all genetic backgrounds and conditions, PhoP D53A strains failed to induce Pho regulon expression, despite normal growth, whereas wild-type *phoP* strains showed induction patterns consistent with Figure 3.

Together, these results demonstrate that elevated PhoP abundance in a *ccpA* mutant is insufficient to drive Pho regulon activation. Instead, Pho regulon induction strictly depends on the phosphorylation state of PhoP, indicating that transcriptional activation is controlled at the level of PhoP phosphorylation rather than protein abundance.

### 3.3. The PhoR-Independent Pho Induction Is Independent of Pi Concentration in the phoR ccpA Mutant Strain

To determine the temporal and quantitative dynamics of *phoB*-P_V_-*lacZ* expression in the *phoR ccpA* background, growth and reporter activity were analyzed in low- and high-phosphate defined media supplemented with glucose (LPDMglu and HPDMglu). Wild-type and *phoR ccpA* strains were monitored during growth under both conditions (Figure 4A,B). In LPDMglu, the wild-type strain exhibited the expected classical Pho response, with *phoB*-P_V_-*lacZ* induction occurring at the transition phase (T0), coinciding with phosphate depletion to approximately 0.1 mM Pi, as previously reported [9,31]. In contrast, the *phoR ccpA* mutant displayed low but constitutive *phoB*-P_V_-*lacZ* expression throughout growth without a defined induction phase (Figure 4A). Under HPDMglu conditions, the *phoR ccpA* mutant maintained a similar level of *phoB*-P_V_-*lacZ* expression as observed in LPDMglu, whereas the wild-type strain showed no detectable induction across the growth curve (Figure 4B).

Together, these data confirm that PhoR-independent Pho regulon expression in the *phoR ccpA* mutant is uncoupled from external inorganic phosphate concentration and is instead maintained in a glucose-dependent, Pi-insensitive manner, consistent with the plate-based assays shown in Figure 1 and Figure 3.

### 3.4. Pho Regulon Gene Expression in the phoR ccpA Background Is Strictly Dependent on the Phosphorylation State of PhoP

To determine the in vivo phosphorylation state of PhoP in the *phoR ccpA* mutant, we employed Phos-tag^TM^ acrylamide SDS-PAGE combined with Western blot analysis. Prior to in vivo application, we validated whether Phos-tag^TM^ gels could resolve phosphorylated and non-phosphorylated PhoP using an in vitro phosphotransfer system consisting of autophosphorylated GST–PhoR~P and PhoP. Autoradiographic detection and immunoblot analysis confirmed the presence of a slower-migrating PhoP species corresponding to PhoP~P (Figure 5A–C). Western blotting with PhoP-specific antibodies detected two distinct bands: unphosphorylated PhoP and a mobility-shifted PhoP species consistent with PhoP~P, confirming that Phos-tag^TM^ SDS-PAGE coupled with immunodetection can reliably discriminate PhoP phosphorylation states.

To assess PhoP phosphorylation in vivo, wild-type and *phoR ccpA* strains were grown in LPDMglu and analyzed over the growth curve. Phos-tag^TM^ analysis (Figure 6A) revealed that PhoP~P was detectable in the wild-type strain between T1 and T5. In contrast, the *phoR ccpA* mutant showed detectable PhoP~P from T-3 to T2 despite the absence of PhoR, confirming that PhoP phosphorylation occurs in vivo independently of its cognate histidine kinase. Alkaline phosphatase (APase) activity confirmed classical Pho induction in the wild-type strain beginning at transition phase (T0), whereas the *phoR ccpA* mutant exhibited an early basal activity that gradually declined over time (Figure 6B). Quantitative analysis of APase activity was consistent with the percentage of phosphorylated PhoP profiles observed in both the wild-type and *phoR ccpA* mutant strains, further supporting the correlation between phosphorylated-to-unphosphorylated PhoP ratios and Pho regulon activation in vivo (Figure 6C).

Quantification of phosphorylation levels showed that the proportion of phosphorylated PhoP correlated with APase activity more closely than absolute PhoP~P abundance. Although the absolute level of PhoP~P in the *phoR ccpA* mutant at T−3 was approximately twofold higher than that in wild-type at T5, APase activity was ~3.2-fold lower, indicating that Pho regulon output is not determined by PhoP~P quantity alone but by the fraction of phosphorylated PhoP relative to total PhoP.

These findings suggest that Pho regulon expression is governed by the phosphorylation state of PhoP rather than absolute PhoP~P levels, raising the possibility that excess unphosphorylated PhoP may influence transcriptional output in vivo. This is consistent with previous observations that overexpression of PhoP from a multicopy plasmid in a wild-type background reduces Pho regulon induction and with in vitro data showing that both PhoP and PhoP~P can bind Pho regulatory DNA sequences, albeit with different affinities.

### 3.5. In the Absence of PhoR and CcpA, YycG May Act as a Non-Cognate Kinase Mediating PhoP Phosphorylation In Vivo

The observed PhoR-independent Pho regulon activation prompted the question of which phosphoryl donor is responsible for PhoP phosphorylation in the *phoR ccpA* mutant. One possibility is transfer from small-molecule phosphodonors, such as acetyl phosphate or carbamoyl phosphate, which are known to phosphorylate response regulators in vivo and in vitro, including CheY in Escherichia coli [35], and the PhoP orthologue PhoB [36,37]. However, our previous in vitro analyses indicated that PhoP phosphorylation by small-molecule phosphodonors is inefficient or undetectable [22], suggesting that a protein kinase is more likely responsible in vivo.

Based on sequence and structural classification of histidine kinases, Fabret et al. grouped *B. subtilis* HKs according to conservation surrounding the phosphorylated histidine and transmembrane architecture [38]. Among these, ResE and YycG share similarities with PhoR, making them plausible candidates for non-cognate phosphorylation of PhoP. Although ResE was considered due to reported temporal coordination between ResD expression and Pho induction (Abdel-Fattah et al., unpublished), disruption of *resE* did not affect *phoB*-P_V_-*lacZ* expression in wild-type or mutant backgrounds, and PhoP phosphorylation in the *phoR ccpA* strain preceded both Pho and Res induction in wild-type cells (Figure 6A,B), collectively arguing against ResE involvement. In contrast, YycG emerged as a stronger candidate due to its essential role in cell wall homeostasis and its regulatory timing, as YycF expression declines at the transition phase [39]. Because YycG is essential, direct genetic disruption was not feasible. Instead, co-immunoprecipitation experiments were performed in *phoR ccpA* cells grown under high-phosphate conditions (HPDMglu), where maximal PhoP~P levels were observed (T_-3_ to T_-1_; Figure 6A). Following formaldehyde crosslinking, immunoprecipitation with PhoP-specific antibodies revealed co-precipitation of YycG, with a complex migrating at ~150 kDa (Figure 7). The observed molecular weight is consistent with multiple possible stoichiometries, including PhoP dimer–YycG monomer or higher-order assemblies. Several attempts were made to identify the components of the 150-kDa band by mass spectrometry; however, these efforts were unsuccessful. We speculate that extensive formaldehyde crosslinking may have interfered with efficient protein recovery and peptide identification, a known limitation of crosslinking-based protein complex analyses. Therefore, we cannot exclude the presence of additional protein component(s) within the 150-kDa species.

To further assess functional phosphotransfer, in vitro assays comparing PhoR and YycG were performed. As expected, rapid and efficient phosphotransfer from PhoR~P to PhoP occurred within seconds (Figure 8A). In contrast, YycG~P was capable of transferring phosphate to PhoP, but with markedly reduced rate and efficiency (Figure 8B). Together, these results support YycG as a candidate non-cognate histidine kinase capable of phosphorylating PhoP in the *phoR ccpA* background, although with substantially lower catalytic efficiency than the cognate kinase PhoR and independently of phosphate limitation.

These findings are consistent with the broader concept of two-component system crosstalk, which is generally suppressed in vivo by cognate phosphatase activity and kinetic insulation despite frequent in vitro detectability [15]. Previous reports have suggested potential cross-regulation between Pho and Yyc systems [17], including indirect evidence linking PhoR-dependent phosphorylation events to YycF-regulated gene expression. However, our data extend these observations by demonstrating that PhoP phosphorylation can occur in vivo via YycG under conditions of combined PhoR and CcpA inactivation, thereby linking carbon catabolite control with envelope stress signaling and Pho regulon activation.

## 4. Discussion

### 4.1. CcpA Functions as an Indirect Regulator of PhoP Signaling Specificity

**Figure 9:**
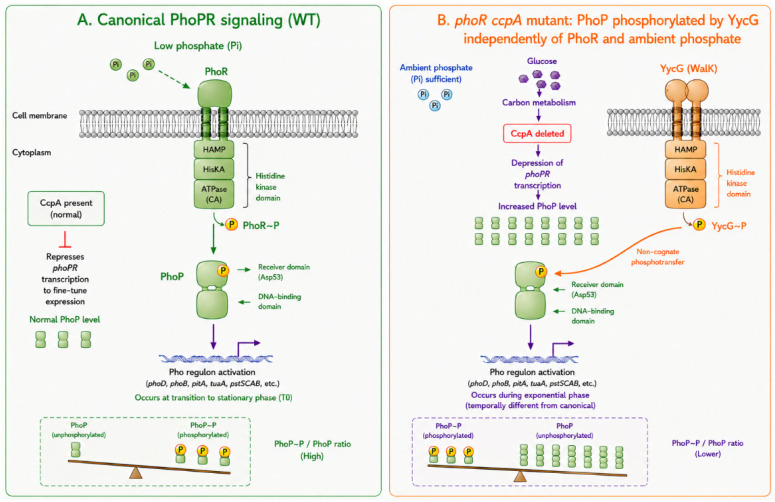
Proposed model illustrating compensatory phosphorylation of PhoP by the non-cognate histidine kinase YycG in the absence of PhoR. (**A**) Under phosphate starvation conditions in the wild-type strain, the cognate sensor kinase PhoR phosphorylates PhoP, generating a high PhoP~P/PhoP ratio that activates Pho regulon expression primarily during the transition to stationary phase. Carbon catabolite repression mediated by CcpA limits *phoPR* transcription, thereby maintaining appropriate PhoP abundance and signaling homeostasis. (**B**) In the *phoR ccpA* mutant, deletion of *ccpA* derepresses *phoPR* transcription in the presence of glucose, resulting in approximately threefold higher cellular PhoP levels. Under these conditions, the essential histidine kinase YycG (WalK) phosphorylates PhoP, enabling Pho regulon expression during exponential growth. The model further emphasizes that Pho regulon temporal output is determined by the balance between phosphorylated and unphosphorylated PhoP rather than by PhoP abundance alone.

The present study reveals an unexpected role for CcpA in maintaining the fidelity of phosphate-responsive signal transduction. Previous work established that CcpA directly represses transcription from the *phoP* promoter P_A6_ in the presence of preferred carbon sources, thereby linking carbon metabolism to phosphate regulation [7]. Our findings extend this model by demonstrating that loss of CcpA not only increases PhoP abundance (Figure 2) but also creates conditions that permit activation of the Pho regulon independently of the cognate sensor kinase PhoR (Figure 1, Figure 3 and Figure 4, and the model in Figure 9). Importantly, elevated PhoP levels alone were insufficient to activate Pho-dependent transcription, as strains carrying the non-phosphorylatable PhoP D53A mutant failed to induce Pho regulon expression despite increased PhoP abundance. These observations indicate that CcpA influences phosphate signaling through a mechanism that extends beyond transcriptional regulation of *phoPR* and suggest that control of PhoP concentration may be critical for preserving signaling specificity within the cellular signaling network.

A plausible explanation is that elevated intracellular PhoP increases the probability of low-efficiency interactions with non-cognate histidine kinases that would normally be negligible under physiological conditions. Thus, by limiting PhoP accumulation, CcpA may indirectly suppress inappropriate phosphorylation events and maintain insulation between two-component regulatory systems. This concept expands the traditional view of CcpA as a metabolic regulator and suggests an additional role in safeguarding the specificity of bacterial signal transduction. However, the mechanism linking glucose availability to compensatory PhoP phosphorylation by YycG remains an important subject for future investigation.

### 4.2. Pho Regulon Output Depends on the Balance Between Phosphorylated and Unphosphorylated PhoP

A major finding of this study is that Pho regulon activation correlates more closely with the proportion of phosphorylated PhoP than with the absolute abundance of PhoP or PhoP~P (Figure 5 and Figure 6). Although phosphorylated PhoP was detectable in both wild-type and *phoR ccpA* strains, transcriptional output did not scale directly with total PhoP~P levels. Instead, the ratio of phosphorylated to unphosphorylated PhoP appeared to be a more reliable predictor of Pho regulon activity.

The gradual decline in APase activity observed in the *phoR ccpA* mutant upon entry into stationary phase may be explained by changes in the balance between phosphorylated and unphosphorylated PhoP. As shown in Figure 6A, the *phoR ccpA* mutant accumulates both phosphorylated PhoP (PhoP~P) and elevated levels of unphosphorylated PhoP during this stage of growth. We previously demonstrated that excess PhoP, particularly PhoP~P but also, to a lesser extent, unphosphorylated PhoP, can repress transcription from certain Pho regulon promoters containing PhoP-binding sites within coding regions or when present at high concentrations in in vitro transcription reactions [31,32]. Therefore, we propose that the accumulation of unphosphorylated PhoP, together with an altered PhoP~P/PhoP ratio during the transition to stationary phase, contributes to repression of alkaline phosphatase transcription, resulting in the gradual decline in APase activity observed in the *phoR ccpA* mutant.

These findings suggest that response regulator signaling should be considered in terms of phosphorylation equilibrium rather than absolute protein abundance. Similar principles may apply broadly to other bacterial two-component systems where both phosphorylated and unphosphorylated forms interact with regulatory DNA targets.

### 4.3. Non-Cognate Phosphorylation Reveals Functional Crosstalk Between the PhoPR and YycFG/WalRK Signaling Systems

Two-component systems are generally regarded as highly insulated signaling pathways that preserve specificity through selective kinase–response regulator interactions [14,15]. Nevertheless, increasing evidence indicates that non-cognate phosphorylation can occur under certain physiological or genetic conditions [16]. Our results provide direct evidence supporting such crosstalk between the PhoPR and YycFG signaling systems.

Several lines of evidence identify YycG as a likely source of PhoP phosphorylation in the absence of PhoR. First, PhoP phosphorylation was detected in vivo in *phoR ccpA* cells (Figure 6), demonstrating that an alternative phosphodonor exists. Second, co-immunoprecipitation experiments revealed physical association between PhoP and YycG (Figure 7). Third, in vitro phosphotransfer assays demonstrated that phosphorylated YycG can transfer phosphate to PhoP, albeit less efficiently than PhoR (Figure 8). Previous studies reported phosphorylation of the YycF response regulator by PhoR during phosphate limitation [17], suggesting that information flow between these two systems may be bidirectional.

The present work expands the known network of interactions between the phosphate-responsive (PhoPR) and cell wall-responsive (YycFG/WalRK) regulatory pathways. During phosphate starvation in the wild-type strain, PhoP~P represses the synthesis of phosphate-rich glycopolymer wall teichoic acid (WTA) through the *tagA* and *tagD* promoters while activating synthesis of the phosphate-free glycopolymer teichuronic acid from the *tuaA* promoter [40]. PhoP~P also induces the secreted phosphodiesterases PhoD [5,21] and GlpQ [41], which scavenge phosphate from WTA and facilitate its replacement with teichuronic acid. Our findings suggest that, by increasing PhoP expression and disrupting the balance between phosphorylated and unphosphorylated PhoP during phosphate starvation, *ccpA* deletion could alter the extent or timing of these cell wall remodeling events and consequently influence YycFG signaling. However, this hypothesis remains speculative and requires direct experimental validation to determine how *ccpA* disruption affects cell wall architecture and YycFG signaling.

### 4.4. Biological and Therapeutic Implications of Alternative PhoP Phosphorylation

Because phosphate limitation is encountered in macrophage phagosomes, epithelial-cell vacuoles, biofilms, and chronically infected tissues, where it promotes persistence, stress adaptation, and antimicrobial tolerance [42,43,44], the signaling plasticity described here may represent a mechanism by which bacteria maintain phosphate-responsive gene expression when canonical PhoPR signaling is impaired. Given the high conservation of PhoPR/PhoB-PhoR systems among Gram-positive and Gram-negative bacteria, our observations in *B. subtilis* may, therefore, provide broader insight into persistence-associated regulatory mechanisms in bacterial pathogens.

Our results also have implications for the development of anti-virulence strategies targeting phosphate-responsive signaling. Previous studies have explored phosphate supplementation to prevent gut-derived sepsis caused by virulent *Pseudomonas aeruginosa* following surgery [45] and inhibition of *Mycobacterium tuberculosis* PhoR autophosphorylation using tamoxifen derivatives [46]. However, our finding that PhoP can be phosphorylated by a non-cognate kinase independently of both its cognate kinase and ambient phosphate availability suggests that strategies targeting PhoR alone, or relying solely on phosphate supplementation to suppress PhoPR signaling, may not completely eliminate Pho regulon activity. Instead, targeting the common downstream function of PhoP, namely, its interaction with target promoters, may represent a more effective approach for disrupting phosphate-responsive adaptation by overcoming the regulatory plasticity conferred by alternative kinase inputs. Consistent with this concept, we previously identified mutations in the PhoP DNA-binding domain that impair Pho regulon activation [47]. Likewise, naturally occurring mutations affecting the PhoP DNA-binding domain in avirulent *Mycobacterium tuberculosis* strains abolish ESAT-6 secretion and impair antigen-specific T-cell recognition [48], demonstrating that disruption of PhoP DNA-binding activity can profoundly attenuate bacterial virulence. These observations further support PhoP as an attractive downstream target for anti-virulence strategies.

### 4.5. Limitations and Future Directions

Although the data presented here support YycG as a candidate non-cognate kinase for PhoP, definitive confirmation of its physiological contribution remains challenging because YycG is essential for viability and cannot be readily eliminated genetically. Future studies employing conditional depletion systems, kinase-dead variants, or phosphoproteomic approaches may help quantify the relative contribution of YycG-mediated phosphorylation in vivo.

In addition, the possibility that other histidine kinases participate in PhoP phosphorylation cannot be excluded. The bacterial signaling network contains numerous potential kinase–response regulator interactions that may become detectable under conditions of altered protein abundance or disrupted regulatory control. Systematic analysis of kinase specificity under varying physiological conditions will be necessary to determine whether the PhoP–YycG interaction represents a unique adaptation or part of a broader network of stress-responsive signaling crosstalk. Moreover, given the predicted molecular masses of YycG (~70 kDa) and PhoP (~27.5 kDa), the molecular weight of the PhoP–YycG crosslinked complex is estimated to be ~150 kDa (Figure 7), which exceeds that expected for a simple 1:1 PhoP–YycG complex. Therefore, the detected species may represent a higher-order complex containing additional protein component(s) and/or oligomeric forms of PhoP or YycG that are stabilized by formaldehyde crosslinking. Accordingly, we interpret the co-immunoprecipitation data as evidence that PhoP and YycG are associated within the same protein complex under the conditions tested. However, we cannot exclude the presence of additional protein component(s) within the 150 kDa protein complex.

Overall, the present study identifies a previously unrecognized connection between carbon catabolite repression, phosphate starvation signaling, and two-component system specificity, providing new insight into how bacterial regulatory networks maintain function under conditions that challenge canonical signal transduction pathways.

## 5. Conclusions

This study demonstrates that activation of the phosphate starvation response in *Bacillus subtilis* is not exclusively dependent on the cognate sensor kinase PhoR. Instead, disruption of carbon catabolite repression permits phosphorylation of PhoP by the non-cognate histidine kinase YycG, thereby maintaining partial Pho regulon activation independently of both PhoR and environmental phosphate availability. Our findings further show that Pho regulon activity is governed not only by PhoP abundance but also by the balance between phosphorylated and unphosphorylated PhoP, highlighting the importance of response regulator phosphorylation dynamics in determining transcriptional output.

These findings reveal an unexpected level of regulatory plasticity within bacterial two-component signaling networks and demonstrate functional crosstalk between the PhoPR and YycFG/WalRK systems. Such compensatory phosphorylation provides a mechanism by which bacteria may preserve phosphate-responsive gene expression when canonical signaling is compromised, potentially enhancing adaptation to nutrient-limited environments.

From a translational perspective, our results suggest that alternative kinase inputs could limit the effectiveness of therapeutic strategies directed solely at sensor histidine kinases. Instead, targeting the common downstream function of the response regulator, particularly PhoP/PhoB DNA binding, may represent a more robust approach for disrupting phosphate-responsive adaptation. Although the physiological relevance of this mechanism remains to be established in pathogenic bacteria, the conservation of PhoPR/PhoB-PhoR signaling suggests that similar regulatory plasticity may contribute to bacterial persistence and antimicrobial tolerance during infection. Collectively, this work provides a conceptual framework for understanding signal integration in bacterial regulatory networks and establishes a foundation for future studies exploring non-cognate phosphorylation as a potential target for anti-virulence therapy.

## Figures and Tables

**Table 1:** List of bacterial strains, plasmids, and primers used in this study.

Strains/Plasmids/Primers	Relevant Genotype or Description	Source/Reference
***E. coli***		
DH5α		Lab stock
BL21(DE3)		Novagen, Chicago, IL, USA
BL21(DE3)/pLysS		Novagen, Chicago, IL, USA
***B. subtilis***		
JH642	*pheA1, trpC2*	J. A. Hoch
MH6018	*pheA1, trpC2, ccpA*::Tn*917*Spc^r^	[7]
MH5124	*pheA1, trpC2, phoRΔBal*l::Tet^r^	[18]
MH696	*pheA1, trpC2, amyE*::*phoB* P_V_*-lacZ:* Cm^r^	[19]
MH4040	*pheA1, trpC2, amyE*::*phoA-lacZ:* Cm^r^	[20]
MH5562	*pheA1, trpC2, amyE*::*phoP40-lacZ:* Cm^r^	[6]
MH5801	*pheA1, trpC2, amyE*::*phoD*-*lacZ:* Cm^r^	[21]
MH9235	*pheA1, trpC2, phoRΔBal*l::Tet^r^*amyE*::*phoB* P_V_*-lacZ:* Cm^r^	This study
MH9236	*pheA1, trpC2, ccpA*::Tn*917*Spc^r^*amyE*::*phoB* P_V_*-lacZ:* Cm^r^	This study
MH9237	*pheA1, trpC2, ccpA*::Tn*917*Spc^r^*phoRΔBal*l::Tet^r^*amyE*::*phoB* P_V_*-lacZ:* Cm^r^	This study
MH7402	*pheA1, trpC2 mdh*::Km^r^*phoPD53A amyE*::*phoB* P_V_*-lacZ:* Cm^r^	This study
MH7407	*pheA1, trpC2 ccpA*::Tn*917*Spc^r^*mdh*::Km^r^*phoPD53A amyE*::*phoB* P_V_*-lacZ:* Cm^r^	This study
MH7408	*pheA1, trpC2 ccpA*::Tn*917*Spc^r^*mdh*::Km^r^*phoPD53A phoRΔBal*l::Tet^r^*amyE*::*phoB* P_V_*-lacZ:* Cm^r^	This study
MH7414	*pheA1, trpC2 mdh*:: Km^r^*phoPD53A phoRΔBal*l::Tet^r^*amyE*::*phoB* P_V_*-lacZ:* Cm^r^	This study
MH9500	*pheA1, trpC2, phoRΔBal*l::Tet^r^*amyE*::*phoP40-lacZ:* Cm^r^	This study
MH9501	*pheA1, trpC2, ccpA*::Tn*917*Spc^r^*amyE*::*phoP40-lacZ:* Cm^r^	This study
MH9502	*pheA1, trpC2, ccpA*::Tn*917*Spc^r^*phoRΔBal*l::Tet^r^*amyE*::*phoP40-lacZ:* Cm^r^	This study
MH9503	*pheA1, trpC2, phoRΔBal*l::Tet^r^*amyE*::*phoD*- *lacZ:* Cm^r^	This study
MH9504	*pheA1, trpC2, ccpA*::Tn*917*Spc^r^*amyE*::*phoD*-*lacZ:* Cm^r^	This study
MH9505	*pheA1, trpC2, ccpA*::Tn*917*Spc^r^*phoRΔBal*l::Tet^r^*amyE*::*phoD*- *lacZ:* Cm^r^	This study
MH9506	*pheA1, trpC2, phoRΔBal*l::Tet^r^*amyE*::*phoA-lacZ:* Cm^r^	This study
MH9507	*pheA1, trpC2, ccpA*::Tn*917*Spc^r^*amyE*::*phoA-lacZ:* Cm^r^	This study
MH9508	*pheA1, trpC2, ccpA*::Tn*917*Spc^r^*phoRΔBal*l::Tet^r^*amyE*::*phoA-lacZ:* Cm^r^	This study
**Plasmids**		
pWL32	pET16b::*phoP*, Amp^r^	[22]
pES5	phoR gene in the *Sma*I site of pJM103	[18]
pDG782	*S. faecalis aphA3* gene (Kan^R^) inserted into the *Cla*I site of pMTL22	T. Masdek
pLS21	pGEX-2T GST-* *phoR* fusion, Amp^r^	[23]
pJT05	pET28a::yycGc, Km^r^	[24]
pUC18	Cloning vector: Amp^r^	[25]
pUC18tet	pUC18 with *tet*; Amp^r^ Tet^r^	[26]
pHT4*phoPR*	pHT315::*mdh phoPR polA*;	T. Masdek
pAT110	Vector for a mutant generation; Em^r^	[27]
pBK1	Blunt-ended 1.36 kb *Sma*I/*Bss*HII *phoP* gene fragment from pHT4*phoPR* subcloned into the *SmaI* site of pUC18	This study
pBK2	Introduced a single base pair mutation at Asp53 of the *phoP* gene in pBK1; Amp^r^	This study
pBK3	Blunt-ended Bpu10I/SspI *tet* gene fragment from pUC18tet subcloned into the *Eco*47II site of pBK2; Amp^r^ Tet^r^	This study
**Primers**		
Bk3	5′-GATTTGATTGTGCTTGCTGTGATGCTTCCAAAATTG-3′	
Bk4	5′-CAATTTTGGAAGCATCACAGCAAGCACAATCAAATC-3′	

## Data Availability

Data are available upon request from the authors.

## Supplementary Materials

The following supporting information can be downloaded at: https://www.mdpi.com/article/10.3390/biology15141138/s1, File S1: Original images.
