# Supplementary material for: Simultaneous Disruption of Phosphate and Carbon Signaling Regulators Enables Adaptive Gene Expression Through Non-Cognate Phosphorylation of PhoP
Source: Biology (Basel). 2026 Jul 13;15(14):1138. doi: 10.3390/biology15141138 (PMC13403408; doi:10.3390/biology15141138)

Figure 2. Quantitative western blot PhoP in WT and *ccpA* during growth LPDM with glucose (G) or glutamate (glu)

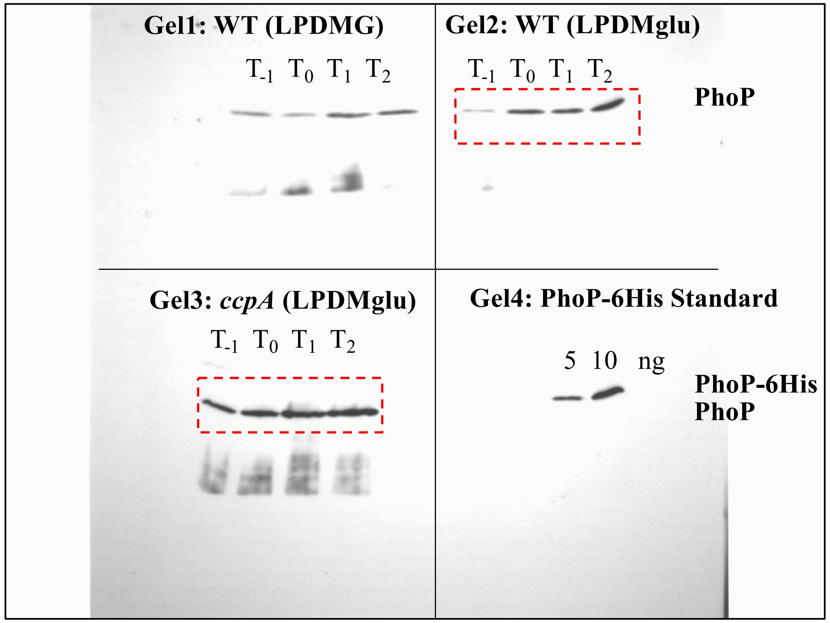

PVDF western blot: anti-PhoP CTD
30 seconds exposure

Figure 2 second experiment:

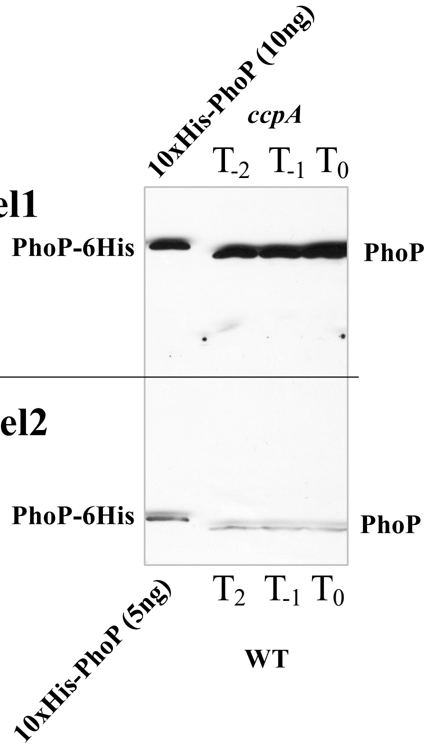

Figure 5: *in vitro* PhoP phosphorylation by **partially digested GST-PhoR?** using γ-^{32}P ATP, reactions run on Phos-tag gel (25 µM, 10% SDS-PAGE), transferred to PVDF, ^{32}P decay, then western blot of PhoP.

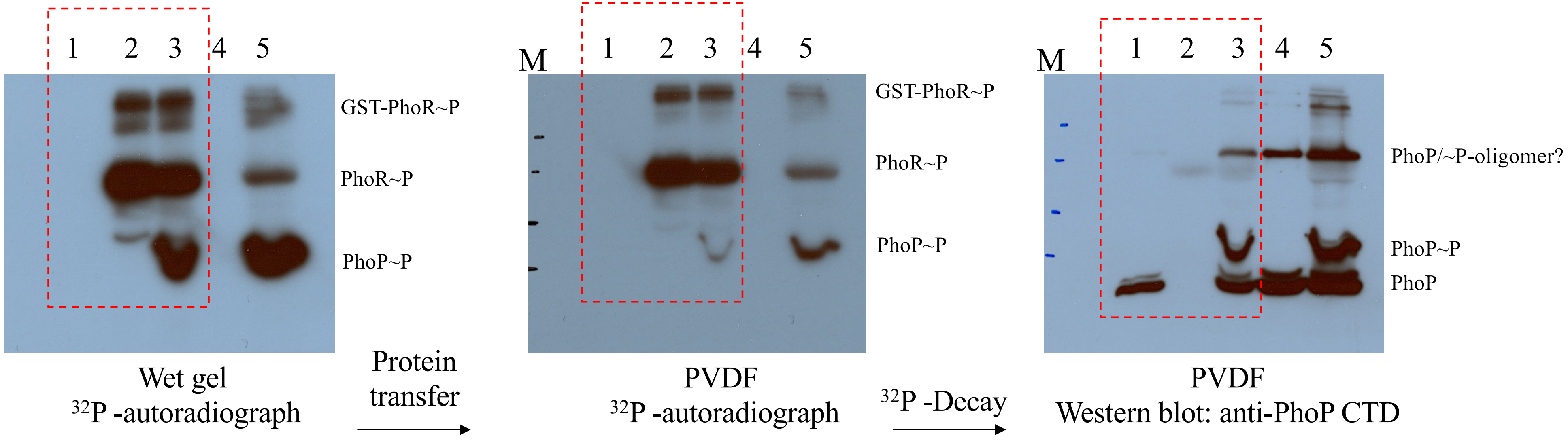

1. PhoP (monomer)
2. Autophosphylated PhoR
3. Autophosphylated PhoR + PhoP (monomer)
4. PhoP (dimer)
5. Autophosphylated PhoR + PhoP (dimer)

Figure 6: Western blot of PhoP in WT, *phoPD53A*, or *ccpA phoR* in LPDMglu samples run on 10% Acrylamide, 5 μM Phos-Tag.

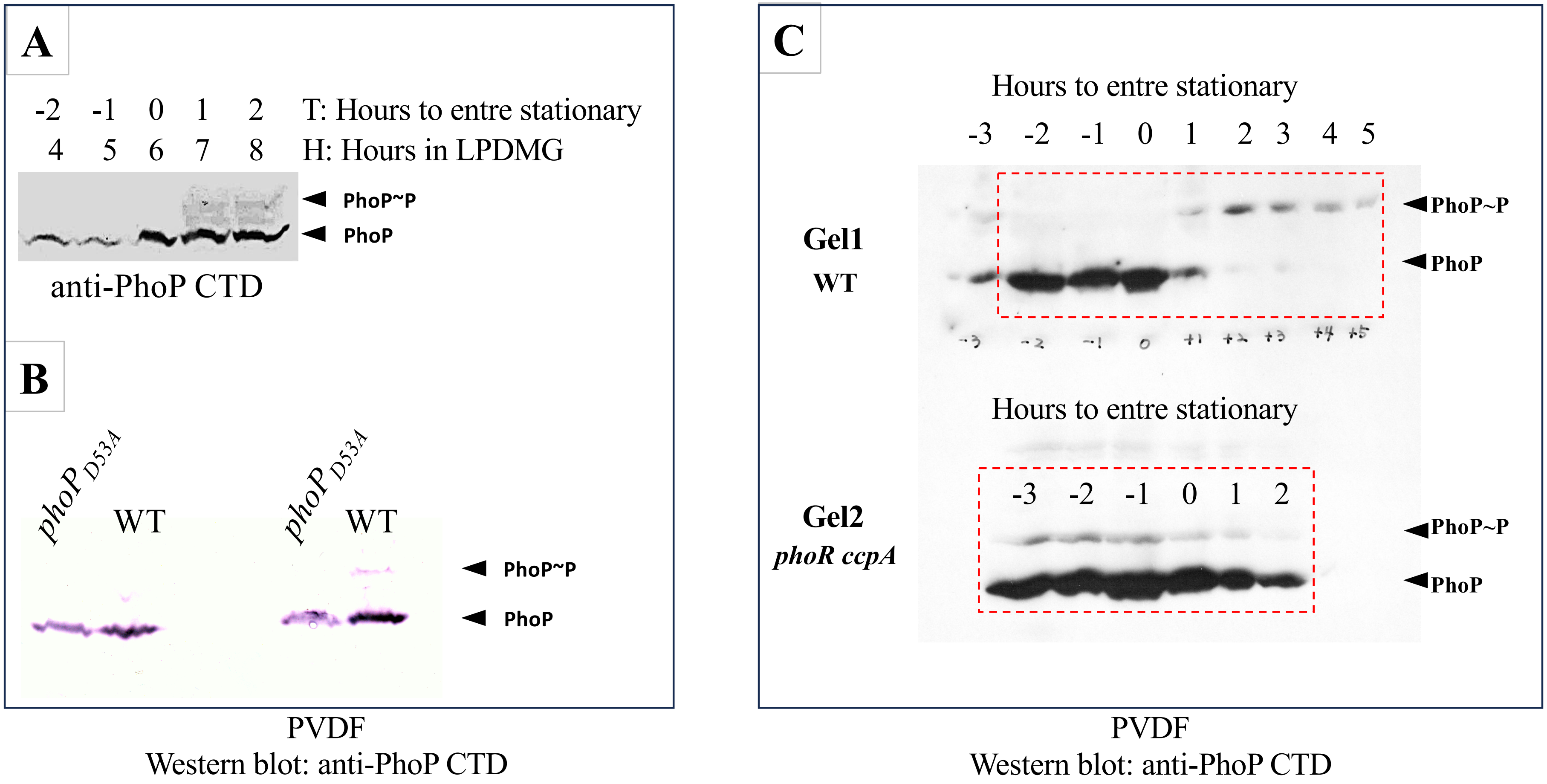

Figure 7: IP of PhoP, run on 10% SDS-PAGE, transfer to PVDF, western blot detection of YycG, strip, then western blot detection of PhoP.

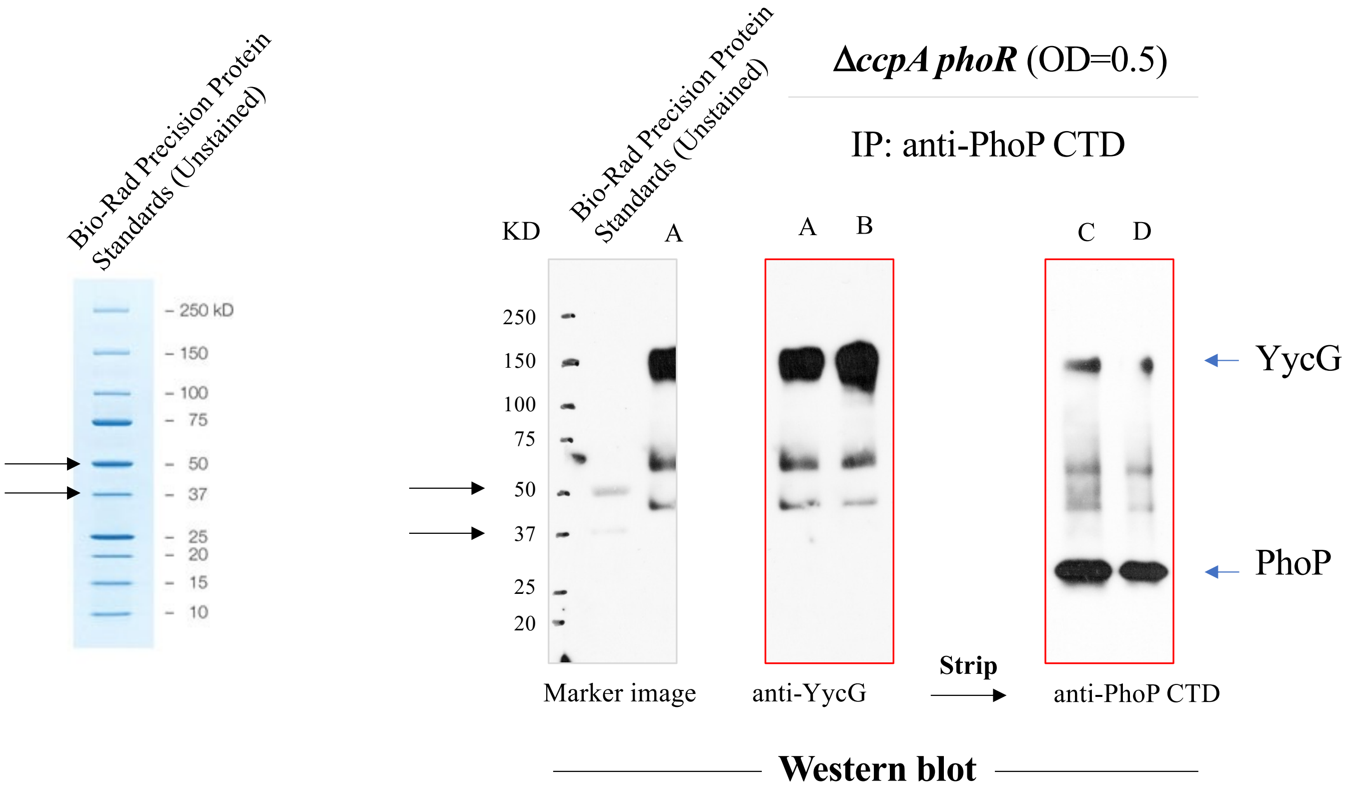

Supplement: Supplementary file 1 [file biology-15-01138-s001.zip › biology-4385042-supplementary File S1.pdf]
